# Squamous Cell Carcinoma Arising from Chronic Osteomyelitis in the Extremities: Treatment Approach and Oncological Outcomes—A Systematic Review of the Literature

**DOI:** 10.1155/2022/2671420

**Published:** 2022-10-10

**Authors:** Mayte Bryce-Alberti, M. R. Gonzalez, Andres Quevedo-Ramirez, Juan Pretell-Mazzini

**Affiliations:** ^1^Facultad de Medicina Universidad Peruana Cayetano Heredia, Lima, Peru; ^2^Escuela de Medicina, Universidad Peruana de Ciencias Aplicadas, Lima, Peru; ^3^Miami Cancer Institute, Division of Orthopedic Oncology, Baptist Health System South FL. Plantation, FL, USA

## Abstract

**Aims:**

In chronic osteomyelitis-derived squamous cell carcinoma, what are the demographic and clinical variables, risk factors associated with worse outcomes, and results of treatment modalities used?

**Methods:**

A systematic review was performed using PubMed and EMBASE. Articles were evaluated for inclusion and exclusion criteria, and for quality analysis. PRISMA guidelines were applied. Demographic and clinical data and therapeutic approaches were presented narratively and in descriptive statistics registered at PROSPERO.

**Results:**

Most patients were male (40/49), trauma was the most common etiology (27/36), and about half of all SCC were in the tibia (25/48). Amputation was the main definitive treatment (42/47). Adjuvant treatments were not analyzed. Well-differentiated SCC accounted for 58.3% (21/36) of all tumors. Bone invasion was described in 82.8% (24/29); recurrence, in 7.7% (3/39); and metastasis, in 7.7% (3/39). Recurrence and metastasis occurred more frequently when bone invasion was present (*p* = 0.578 and *p* = 0.646, respectively). SCC with lymph node involvement showed a higher tendency to metastasize (*p* = 0.377). Compared with limb salvage, amputation was associated with a tendency for less recurrence (*p* = 0.312) and longer survival (*p* = 0.219).

**Conclusions:**

COM-derived SCC mostly occurs after trauma and is usually located in the tibia. Bone invasion is common, and patients predominantly undergo amputation. This treatment is associated with a trend toward higher survival, compared to limb salvage.

## 1. Introduction

Squamous cell carcinoma (SCC) represents the second most prevalent skin cancer globally [1], and it commonly affects Caucasians in areas exposed to sunlight [2, 3]. While cutaneous SCC has an excellent prognosis, a subset of tumors presents metastasis and mortality rates of 3.7% and 2.8%, respectively, [4]. Development of the aggressive variant is commonly unrelated to sun exposure, while a history of a chronic wound is prognostic for recurrence, metastasis, and death [5]. Accordingly, Que et al. [6] reported that scar tissue caused by chronic inflammation elevated metastasis rates to 26%, and Edwards et al. [7] evidenced that tumors occurring in sites of chronic or previous wounds had a 58% chance of recurrence and led to a 48% 5-year mortality. In general, recurrent SCC carries a worse prognosis and a higher risk of spread to regional lymph nodes and distant metastasis, for which clinicopathological risk stratification and early detection of lymph node spread are mandatory [6].

Chronic osteomyelitis (COM) represents a long-lasting and persistent inflammatory process that is associated with a high incidence of infection recurrence [8]. In spite of multiple treatment interventions, the reinfection rate ascends to 20–30% [9]. This predisposes patients to develop sinus tracts, fistulas, and ulcers that result in cutaneous malignant transformation, even after decades of COM diagnosis and management.

Incidence of SCC arising from COM ranges from 0.2–1.7% [10–12] and due to its aggressive nature, early aggressive treatment is recommended [13]. Definite treatment involves amputation or wide tumor excision, although the arrival of new immunotherapeutic agents is promising [10, 14].

COM-related SCC has been intermittently reported in case reports, case series, or small retrospective studies that often fail to establish a true causal relationship between COM and SCC [13, 15–38]. Additionally, available literature reviews [39] on the subject incorporate patient populations that mix specific data from COM-derived SCC with the broader condition of Marjolin ulcer, as well as COM that developed as a complication of a pre-existing SCC. Therefore, this systematic review seeks to analyze the following characteristics in patients with SCC arising from COM: what are the (1) demographic and clinical variables, and treatment modalities of these patients, (2) risk factors associated with worse outcomes, and (3) outcomes of treatment modalities used?

## 2. Research Design and Methods

We followed the PRISMA statement [40] and registered our protocol with the International Prospective Register of Systematic Reviews (PROSPERO CRD42021249825).

### 2.1. Eligibility Criteria

Peer-reviewed original articles; publication in English, Spanish, Portuguese, French, or Italian; development of SCC clearly attributed to COM; surgical treatment including any combination of debridement, excision, resection, and/or amputation was performed or suggested; and case reports and case series. Letters to the editor; (2) studies where COM was an independent finding not associated with SCC; and articles in which individual patient data could not be extracted, were excluded.

### 2.2. Literature Search and Study Selection

**Figure 1:**
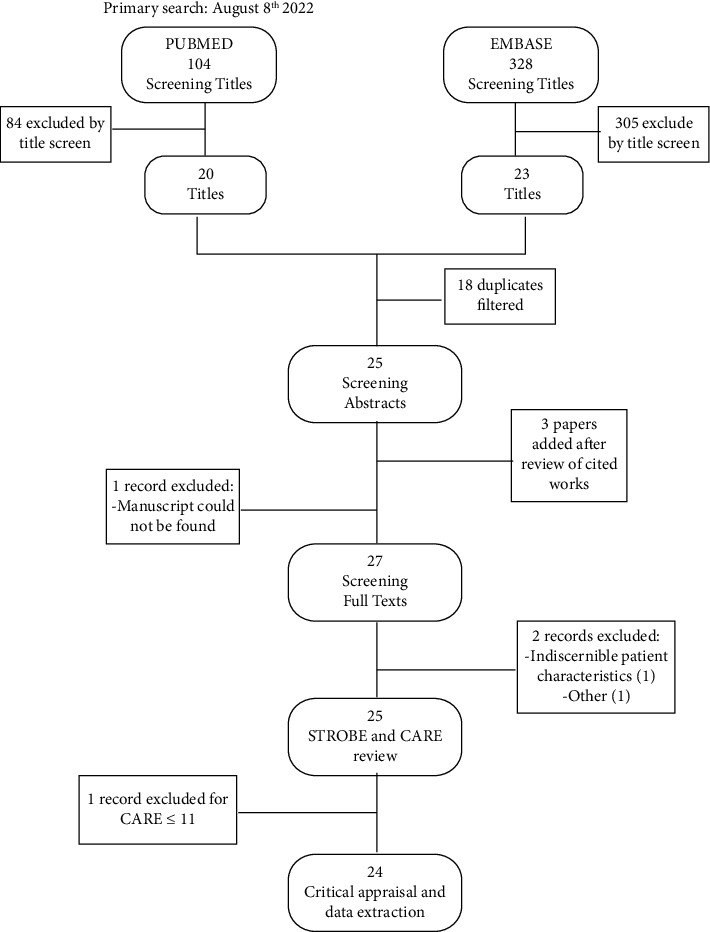
Flowchart for our literature search and selection of relevant articles.

We searched PubMed and EMBASE on August 8^th^ 2022 using the Boolean operators “osteomyelitis” and “squamous cell carcinoma” between January 1, 2010, and August 8, 2022. Two reviewers (M.B-A and M.R.G) independently examined all articles for inclusion. All titles were evaluated for relevance and duplicates were excluded. The resulting abstracts were screened and 27 full texts were evaluated (Figure 1). Bibliographies of the retrieved articles were used to identify other relevant studies.

### 2.3. Assessment of Methodological Quality

Two reviewers (M.B-A and M.R.G) independently used the case reports guidelines (CARE) for case reports and series that individually described patients, and the strengthening the reporting of observational studies in epidemiology statement (STROBE) for case series that pooled patient information [41, 42]. In case of controversy, the senior author (J.P-M) made the final decision.

We utilized 8 of the 13 items of the CARE checklist for the methodological assessment (Supplementary material 1). Each item was scored as well-described (2 points), partially described (1 point), or poorly described (0 points). Articles with >11 points were included. STROBE assessment followed the strategy described by Summers SH et al. (Supplementary material 2) [43]. For this checklist, 10 of the available 22 items were utilized. All items were assigned scores from 0 points to 2 points. Articles with >12 points were included.

### 2.4. Data Collection and Presentation

Two authors (M.B-A and M.R.G) analyzed the included studies using predetermined criteria and extracted the data. COM etiology was classified as trauma, open wound, hematogenous or diabetic foot. Local lymphadenopathy was cataloged as either inflammatory or metastatic and we analyzed lymph node invasion and metastasis as different variables. Sufficient information was provided by the articles included to establish these categories and analyze them separately. Recurrence and metastasis outcomes were evaluated after initial diagnosis and primary intervention. Current status was categorized as no evidence of disease, alive with disease, or dead. The final treatment approach, which was always surgical, was divided into amputation and limb salvage.

Demographic and clinical data and therapeutic approaches were presented narratively and using descriptive statistics. Weighted means and standard deviations were calculated in all available variables. The median and interquartile ranges were used in variables with a low number of observations. Student's *t*-test was used to compare continuous means of 2 groups and Fisher's exact test to assess the difference in proportions of categorical outcomes. Overall 1, 2, and 5-year survival was calculated using the Kaplan–Meier method. Survival difference was assessed using the log-rank test. A *p* ≤ 0.05 was considered statistically significant. Statistical analysis was performed using Stata software (StataCorp LLC, Texas, USA).

## 3. Results

Our study included 24 studies (19 case reports and 5 case series) with 49 patients. Detailed information about clinical characteristics, treatment strategies, and related outcomes is shown in Table 1.

### 3.1. Demographic and Clinical Variables, and Treatment Modalities of Patients with COM-Derived SCC

All articles included in our paper were analyzed in this section. The median age (and IQR) at diagnosis of COM was 29 years ±15.46 and the mean age (and SD) at diagnosis of SCC was 60. ± 11.06 years (Table 2). The mean duration from COM to SCC was 29.69 ± 17.23 years. Most patients (40/49, 81.6%) were male, trauma was the most common etiology (27/36, 75%), the lower limb was involved in most patients (46/48, 93.9%), and almost half of all SCC were in the tibia (25/48, 52.1%). Well-differentiated SCC accounted for 58.3% (21/36) of all tumors. Bone invasion was detected in 82.8% (24/29) of all tumors.

Amputation was performed in most patients (42/47, 89.4%). Above-the-knee amputation and below-the-knee amputation were the most commonly performed surgeries (36.2% (17/47) and 27.7% (13/37), respectively). All patients included in our systematic review did not receive either chemotherapy or radiotherapy; this was done to avoid adding potential confusing factors and restricting our analysis to surgical outcomes.

**Figure 2:**
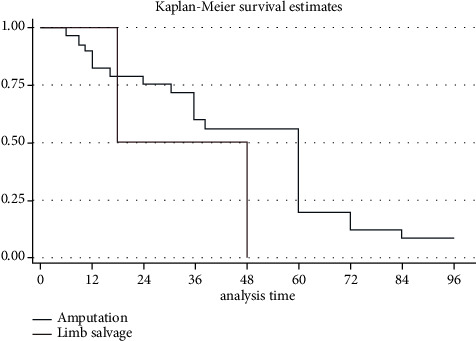
Kaplan–Meier survival curve for all patients with COM-derived SCC was included in our analysis.

Recurrence and metastasis were found in 7.7% (3/39) and 7.7% (3/39) of cases, respectively. At the end of the follow-up period, 73% (27/37) of patients had no evidence of disease, 5.4% (2/37) were alive with disease, and 21.6% (8/37) had died. All 3 patients with metastasis died due to the progression of the disease; the remaining deaths (5/8) occurred due to nonrelated causes in 4 cases, and in the remaining patient, cause of death was not specified. One-year, 2-year, and 5-year overall survival were 84.1%, 74.6%, and 18.4%, respectively, (Figure 2).

### 3.2. Risk Factors Associated with Worse Outcomes

The following articles were analyzed in this section [13, 15–25, 27, 29–34, 37, 38] as they reported risk factors associated with worse outcomes. Although not statistically significant (*p*=0.579), trauma-originated COM-derived SCC was associated with a lower metastasis rate (2/22, 9.09%) compared with other etiologies (1/7, 14.29%) (Table 3). SCC that invaded bone developed recurrence and metastasis in 14.29% (3/21) and 9.52% (2/210) of cases, compared with 0% in patients without invasion (*p*=0.578 and *p*=0.646, respectively). SCC that had lymph node involvement were more likely to develop metastasis than those without it, with a rate of 16.67% (1/6) and 4.35% (1/23), respectively. These results were not statistically significant (*p*=0.377) due to the low occurrence of the events, which significantly limited our analyzed sample size.

### 3.3. Outcomes of Treatment Modalities Used

**Figure 3:**
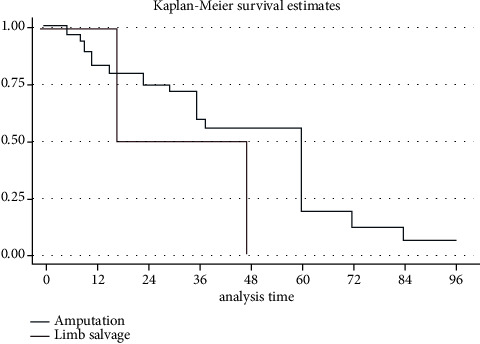
Kaplan–Meier survival curve for patients with COM-derived SCC who underwent amputation or a limb salvage procedure. Log-rank analysis showed no difference between the groups (*p*=0.29).

All articles included in our paper were analyzed in this section. Amputation showed a tendency toward lower recurrence in comparison with limb salvage, with 5.56% (2/36) and 33.33% (1/3) risk of recurrence, respectively, (*p*=0.219) (Table 4). Metastasis rate in the amputation group was 8.33% (3/36) and 0% (0/2) in the limb salvage group; however, only 2 patients who underwent a limb salvage surgery were analyzed in our study. Furthermore, patients who underwent amputation tended to live longer than patients who had a limb salvage procedure, with mean survival times of 39.91 ± 27.63 and 24 ± 21.63 months (Figure 3), respectively. Again, the small sample analyzed for this outcome did not allow the results to be statistically significant (*p*=0.29). Within the amputation subgroup, the most performed treatment modalities were above-the-knee amputation (AKA) and below-the-knee amputation (BKA) with similar mean-survival time, 47.23 ± 31.01 and 45.78 ± 22.03, respectively.

## 4. Discussion

COM is associated with persistent inflammation that can predispose to COM-derived SCC [10–12]. Identifying aggressive variants can provide a better guide for management and follow-up [13, 44]. In this study, we described (1) demographic and clinical variables, and treatment modalities of these patients, (2) risk factors associated with worse outcomes, and (3) outcomes of treatment modalities used.

This study has limitations. There was a lack of standardized information regarding patients' characteristics and treatment approaches. Second-handed analysis of information common in systematic reviews relies on the interpretation of data rather than on acquisition of it; as such, the risk of selection bias is high. Treatment approaches were selected in each case at the discretion of the surgeon, who tends to improve the appearance of outcomes and de-emphasize related complications. Inclusion and exclusion criteria used by the included articles' authors could not be controlled, adding additional limitations to the analysis conducted in our study. Publication bias should also be considered in this study since cases with poor outcomes might be overrepresented in the literature.

Given the nature of COM-derived SCC, most patients in this systematic review presented with tumoral lesions in the lower limb (93.9%). This finding contrasts with the overall incidence of lower limb cSCC (13%) [3] but coincides with Jiang et al. [39]. In accordance with the aforementioned study [39], our study also registers trauma as the most frequent COM etiology (75%) and the tibia as the most affected bone (52.1%). Most cSCC cases are amenable to surgery alone [45] and most of them have an excellent prognosis following resection [46]. Surgery has traditionally been used to treat COM-derived SCC, in which amputation has been the primary management strategy for decades. In our study, most patients underwent amputation (89.4%). As previously stated, in spite of multiple surgical and nonsurgical management strategies of COM, the reinfection rate ascends to 20–30% [9]; thus, although aggressive, amputation allows for the eradication of both infection and malignancy [47]. This may explain why recurrence within our patient population was low (7.7%, 3/39). Depth of invasion in cSCC has traditionally been reported as Breslow thickness, measured from the granular layer of the epidermis, or if the surface is ulcerated, from the base of the ulcer to the deepest point of invasion [48]. Our study demonstrates that evaluating tumor anatomic depth is particularly important in COM-derived SCC given that most patients presented with bone invasion (82.8%). Nodal and distant-organ involvement is rare, as cSCC is mostly a localized neoplasm [48]. We report higher metastatic lymphadenopathy (12.9%) compared to other studies of cSCC (1.5–5.2%) [48–53]. A small percentage (1%) of patients with cSCC present with distant-organ spread [54]. This value is much lower than the 7.7% of patients (3/39) that developed distant metastasis in our study. In these cases, the route of dissemination is hematogenous and in 15% of them, the process may bypass the lymph nodes [55, 56]. Finally, our mortality frequency was much higher than disease-specific death values in other cSCC studies (1.5–2.8%) [48]. This range may be low due to cSCC not always being identified as the official cause of death.

We encountered risk factors associated with poor outcomes: local recurrence, the extent of tumor differentiation, tumor depth, and lymphovascular involvement. In most cases, cSCC behaves as a localized neoplasm with low metastatic risk [45, 52]. Local recurrence is often the first indicator of aggressive tumor behavior that fosters progression to metastasis and death [46, 57]. Our results showed that trauma-originated COM-derived SCC was associated with a low recurrence (4.35%) and metastatic rate (9.09%). However, still higher than cSCC. There are many studies evaluating associations between histologic differentiation of cSCC and recurrence and/or metastasis [2, 58, 59]. Brantsch et al. [2] showed that poor differentiation in cSCC marked a poorer prognosis, with a local recurrence risk more than 3 times higher than the risk from well-differentiated neoplasms (7% versus 2%) and a metastatic risk approximately double (7% versus 3%) that of well-differentiated cSCC. Conversely, our results showed that well-differentiated tumors were associated with higher percentages of recurrence and metastasis (6.67% and 13.33%, respectively) than poorly differentiated tumors, which showed no recurrence and/or metastasis. This finding is not consistent with general oncologic evidence and may be a result of a reduced sample size. Most studies agree that greater tumor depth is associated with a higher relative risk of local recurrence and metastasis [58–60]. In the largest dataset describing anatomic depth, Karia et al. [58] registered that tumors extending beyond subcutaneous fat were considered high-risk and strongly associated with metastasis (RR [95% CI], 7.0 [2.4–20.3]; *p* > 0.001) and disease-specific death (HR [95% CI], 11.1 [3.4–35.8]; *p* < 0.01). Additionally, Clayman et al. [61] determined that cSCC that extended beyond subcutaneous tissue were more likely to recur. Our findings are in accordance with the aforementioned data, as SCC that invaded bone developed recurrence and metastasis in 14.2% and 9.53% of cases, respectively, compared with 0% in patients without invasion. Although nodal metastasis is rare in cSCC, it significantly affects prognosis when it occurs [48]. We found that SCC that had lymph node involvement were more likely to metastasize (16.67%) than those without lymph node compromise (4.35%). Lymphovascular involvement is a poor prognostic factor in cSCC and is associated with a 7.54 increased risk of metastatic spread if present [62]. Moore et al. [63] reported a hazard ratio of 8.03 (3.88–16.2, *p* < 0.0001) using a crude analysis. The potential benefit of early detection of nodal metastasis has led to an increased interest in sentinel lymph node biopsy [64, 65].

Concerning outcomes of treatment modalities used, most cases of SCC have an excellent prognosis following surgical excision [46]. This can be evidenced in our results as amputation was associated with lower recurrence (5.56%) compared to limb salvage (33.33%). This study also showed that patients, who underwent amputation lived longer (39.91 ± 27.63 months) than patients, who had a limb salvage procedure (24 ± 21.63 months). Most of our pooled patients underwent an AKA or a BKA.

Interestingly, none of our collected cases reported treatment with monoclonal antibodies such as Cemiplimab. This human IgG4 monoclonal antibody is directed against PD-1, leading to T cell inactivation and enhancement of the immune system's antineoplastic response. [66] PD-1 represents an immune checkpoint that malignant cells activate to down-regulate the immune system and avoid destruction. [67] PD-1 blockade is particularly effective in tumors with high mutation rates such as melanoma, nonsmall cell lung cancer, and cSCC.

Concerning cSCC, immunotherapy is the only approved treatment for a metastatic or locally advanced disease that cannot be treated with curative surgery or radiation. [48, 68] The expansion cohorts of a phase 1 study in patients with locally advanced or metastatic cSCC, reported a response to this drug in 50% of the group (CI 95% 30–70), and in 47% (CI 95% 34–61) of a phase 2 study cohort of patients with metastatic disease. The duration of response exceeded 6 months in 57% of those that exhibited one. [69] The role of anti-PD-1 in the adjuvant or neo-adjuvant therapy is still being evaluated in ongoing trials. Additionally, platin-based chemotherapy and anti-EGFR immunotherapy are being explored as possible second-line treatments; thus, expanding on options for patients with advanced disease or those in whom limb salvage is not possible. [68].

## 5. Conclusions

COM-derived SCC mostly occurs in patients having a history of post-traumatic COM. The tumor favors males and the tibia. Although most SCC is well-differentiated, bone invasion is common, and patients predominantly undergo amputation. Despite lack of statistical significance, there is a trend toward higher survival in patients who undergo amputation compared to those with limb salvage procedures. Overall, COM-derived SCC presents worse oncological outcomes than cSCC when compared to published data.

## Figures and Tables

**Table 1:** Clinical characteristics, treatment strategies, and related outcomes of all patients included in this study. *Dx: diagnosis*, SCC*: squamous cell carcinoma*, *BKA: below-the-knee amputation*, *AKA: above-the-knee amputation*, *NA: not available*, *Y: yes*, *N: no*, *M: metastasis*, *I: inflammatory*.

Author and Year	Patient no.	Gender	Age at Dx of SCC	Duration to SCC	Etiology	Site	Previous treatment approaches	Final treatment approach	Follow-up	Recurrence	Metastasis	SCC differentiation	Bone invasion	Lymph node involvement
Abdul (2017) [15]	1	Female	58	8	Open wound	Foot	Partial amputation (distal hallux) + debridement	Ray amputation	9	N	N	NA	Y	N
Akoh (2017) [16]	2	Male	44	28	Trauma	Fibula	Trans metatarsal amputation	BKA	30	N	N	Moderately	Y	Y (M)
Alami (2011) [17]	3	Male	53	25	Trauma	Tibia	NA	AKA	84	N	N	Well	Y	N
	4	Male	52	40	NA	Tibia	NA	AKA	60	N	N	Well	Y	N
	5	Female	49	14	Trauma	Tibia	NA	AKA	72	N	N	Well	Y	N
	6	Male	71	22	Trauma	Tibia	NA	AKA	36	N	N	Well	Y	N
	7	Male	60	36	NA	Femur	NA	AKA	60	N	N	Poorly	N	N
	8	Male	58	26	Trauma	Femur	NA	AKA	38	N	N	Poorly	N	N
	9	Male	38	9	Trauma	Humerus	NA	Treatment refused	NA	NA	NA	Well	Y	N
Altunay (2015) [18]	10	Male	53	6	NA	Foot	Amputation (5th toe)	Treatment refused	2	NA	N	Poorly	N	Y (M)
Aslan (2020) [19]	11	Female	71	60	NA	Tibia	Debridement	Additional debridement	48	Y (48)	N	NA	Y	N
Bernhard (2017) [20]	12	Male	63	20	Trauma	Tibia	NA	AKA	NA	N	N	Poorly	NA	Y (M)
Caruso (2016) [21]	13	Male	69	40	Trauma	Tibia	Wide-margin surgical debridement	BKA	2	Y (2)	N	NA	Y	N
Chagou (2020) [22]	14	Male	47	40	Hematogenous	Tibia + fibula	Sequestrectomy + fistula excision	AKA	NA	N	N	NA	Y	NA
Chiao (2014) [23]	15	Male	74	2	Diabetic foot	Foot	Sequestrectomy + debridement	Forefoot amputation	72	N	N	Well	Y	NA
Hamdani (2017) [24]	16	Male	67	51	NA	Femur	Fistula excision + curettage of bone cavity	Hip disarticulation	24	N	N	Well	Y	NA
Henning (2020) [13]	17	Female	65	NA	Open wound	Foot	Metatarsal head resection (3rd toe)	Complete amputation (3rd toe)	12	N	N	Well	Y	Y (I)
Hwang KT (2012) [25]	18	Male	79	15	Trauma	Tibia	NA	En bloc resection	18	N	N	NA	Y	N
Karasov Yesilada (2013) [26]	49	Male	55	43	NA	NA	NA	Tumor excision	NA	NA	NA	Poorly	NA	NA
Kersh (2010) [27]	19	Male	62	NA	NA	Foot	NA	Amputation (5th digit) + resection (Mohs Qx)	16	N	N	Moderately	N	N
Khaladj (2015) [28]	20	Male	89	0.25	NA	Foot	Partial amputation (3rd toe)	Amputation (3rd toe)	NA	NA	NA	Poorly	Y	NA
Kurihara (2019) [30]	21	Male	69	54	Trauma	Femur	Multiple surgeries	Hip disarticulation	8	N	Y (6)	Well	Y	NA
Lack (2010) [31]	22	Female	66	11	Open wound	Pelvic bone + femur	Debridement + proximal femur resection + ischium partial excision	Hemipelvectomy	2	N	Y (0)	Well	Y	Y (M)
Li (2015) [32]	23	Male	51	13	Trauma	Ankle	NA	Treatment refused	NA	NA	NA	Well	Y	N
	24	Male	61	40	Trauma	Femur	NA	Hip disarticulation	60	N	N	Poorly	NA	N
	25	Female	52	33	NA	Tibia	NA	BKA	60	N	N	Well	Y	N
	26	Female	66	20	NA	Tibia	NA	BKA	60	N	N	Poorly	NA	N
	27	Male	45	30	Trauma	Tibia	NA	BKA	60	N	N	Poorly	NA	N
	28	Male	53	30	Trauma	Tibia	NA	BKA	60	N	N	Poorly	NA	N
	29	Male	52	8	Trauma	Tibia	NA	BKA	60	N	N	Poorly	NA	N
	30	Male	58	50	Trauma	Tibia	NA	BKA	60	N	N	Poorly	NA	N
Monaco (2015) [33]	31	Male	60	10	Open wound	Foot	Debridement + multiple reconstructive surgeries	BKA	10	N	N	Poorly	N	NA
Moura (2017) [34]	32	Male	72	65	Trauma	Femur + tibia + fibula	NA	AKA	96	N	N	NA	NA	Y (I)
	33	Male	63	57	Hematogenous	Tibia	NA	AKA	NA	N	N	NA	NA	N
	34	Male	69	62	Hematogenous	Tibia	NA	AKA	24	N	N	NA	NA	N
	35	Male	49	43	Trauma	Tibia	NA	AKA	84	N	N	NA	NA	N
	36	Male	42	32	Trauma	Tibia	NA	AKA	12	N	N	NA	NA	N
	37	Male	75	36	Trauma	Tibia	NA	AKA	6	N	Y (5)	NA	NA	N
Moyer (2016) [35]	38	Male	70	21	Open wound	Tibia	NA	BKA	12	NA	NA	Well	NA	NA
Peng (2020) [36]	39	Male	59	9	Trauma	Tibia + fibula	NA	AKA	NA	NA	NA	Well	NA	NA
	40	Male	58	40	Trauma	Tibia + fibula	NA	AKA	NA	NA	NA	Well	NA	NA
	41	Male	66	50	NA	Tibia	NA	BKA	NA	NA	NA	Well	NA	NA
Stanger (2015) [37]	42	Male	86	35	Trauma	Tibia	NA	Resection	6	N	NA	NA	NA	NA
Steinrücken (2012) [38]	43	Female	62	40	NA	Humerus	No previous surgery	Transhumeral amputation	36	Y (36)	N	Well	Y	NA
	44	Female	54	12	Trauma	Tibia + fibula	Previous surgeries (including surgical excision)	Amputation (left foot)	12	N	N	Well	Y	NA
	45	Male	59	21	Trauma	Tibia	Previous surgeries (14, including sequestrectomy and debridement)	AKA	6	N	N	Well	Y	NA
	46	Male	52	19	Trauma	Tibia + fibula	Previous surgeries (4, including 2 sequestrectomies)	AKA	36	N	N	Well	Y	NA
	47	Male	56	30	Trauma	Tibia	Previous surgeries (6)	Treatment refused	NA	NA	NA	Well	Y	NA
	48	Male	77	39	Trauma	Tibia	Previous surgeries (3, including sequestrectomy, vancomycin-impregnated spacer)	BKA	NA	N	*N*	Well	Y	NA

Data displayed in columns *Age at diagnosis*, *Duration to SCC* refers to years, and data displayed in all remaining columns refer to months. Data displayed in brackets in the *Recurrence* and *Metastasis* columns refer to months elapsed until the event happened. Data displayed in brackets in the *Lymph node involvement* column refers to the inflammatory or metastatic causes of the lymph node involvement.

**Table 2:** Demographic and clinical characteristics, and treatment modalities for patients with chronic osteomyelitis-derived squamous cell carcinoma. Data displayed with ± symbol refers to the standard deviation, while data in parenthesis refers to the percentage of patients. *IQR: interquartile range, COM: chronic osteomyelitis, AKA: above-the-knee amputation, BKA: below-the-knee amputation.*^*a*^*median value*

Age at diagnosis of SCC (years)		60.9 ± 11.06
Age at diagnosis of COM (years)		29^a^ (IQR: 15.46)
Injury duration (years)		29.69 ± 17.23
Follow-up (mo.)		36^a^ (IQR: 12.60)
Gender (*n* = 49)	Male	40 (81.6%)
	Female	9 (18.4%)
Etiology (*n* = 36)	Trauma	27 (75%)
	Hematogenous	3 (8.3%)
	Open wound	5 (13.9%)
	Diabetic foot	1 (2.8%)
Location (*n* = 49)	Upper limb	2 (4.1%)
	Lower limb	46 (93.9%)
	Pelvis + lower limb	1 (2%)
Site (*n* = 48)	Humerus	2 (4.2%)
	Femur	5 (10.4%)
	Tibia	25 (52.1%)
	Fibula	1 (2.1%)
	Ankle	1 (2.1%)
	Foot	7 (14.6%)
	Tibia + fibula	5 (10.4%)
	Femur + tibia + fibula	1 (2.1%)
	Pelvic bone + femur	1 (2.1%)
Type of treatment (*n* = 47)	Amputation	**42 (89.4%)**
	AKA	17 (36.2%)
	BKA	13 (27.7%)
	Foot amputation	2 (4.3%)
	Ray amputation	2 (4.3%)
	Digit amputation	3 (6.4%)
	Hip disarticulation	3 (6.4%)
	Hemipelvectomy	1 (2.1%)
	Transhumeral amputation	1 (2.1%)
	Limb salvage	**5 (10.6%)**
	Resection	4 (8.5%)
	Excision	1 (2.1%)
Differentiation (*n* = 36)	Well	21 (58.3%)
	Moderately	2 (5.6%)
	Poorly	13 (36.1%)
Bone invasion (*n* = 29)	Yes	24 (82.8%)
	No	5 (17.2%)
Local lymph nodes (*n* = 31)	Yes	6 (19.4%)
	No	25 (80.6%)
Recurrence (*n* = 39)	Yes	3 (7.7%)
	No	36 (92.3%)
Metastases (*n* = 39)	Yes	3 (7.7%)
	No	36 (92.3%)
Current status (*n* = 37)	No evidence of disease	27 (73%)
	Alive with disease	2 (5.4%)
	Dead	8 (21.6%)

**Table 3:** Factors potentially associated with increased risk of recurrence, metastasis, and/or all-cause death. *COM: chronic osteomyelitis, SCC: squamous cell carcinoma.*

	Recurrence	Metastasis	All-cause death
*Etiology COM*			
Trauma	4.35% (1/23)	9.09% (2/22)	25% (5/20)
Others^a^	0 (0/7)	14.29% (1/7)	28.57% (2/7)
*Pvalues*	0.767	0.579	0.607

*SCC differentiation*			
Well	6.67% (1/15)	13.33% (2/15)	13.33% (2/15)
Moderately	0 (0/2)	0 (0/2)	0 (0/2)
Poorly	0 (0/10)	0 (0/11)	10% (1/10)
*Pvalues*	1	1	1

*Bone invasion*			
Yes	14.29% (3/21)	9.52% (2/21)	10% (2/20)
No	0 (0/4)	0 (0/5)	20% (1/5)
*Pvalues*	0.578	0.646	0.504

*Local lymphadenopathy*			
Yes	0 (0/5)	16.67% (1/6)	60% (3/5)
No	8.7% (2/23)	4.35% (1/23)	18.18% (4/22)
*P values*	0.669	0.377	0.091

*Duration from COM to SCC (years)*			
>27	13.64% (3/22)	5.88% (1/17)	30% (6/20)
≤27	0 (0/16)	9.52% (2/21)	12.5% (2/16)
*Pvalues*	0.183	0.581	0.199

^a^other include hematogenous, open wound, and diabetic foot.

**Table 4:** Treatment modalities and associated risk of recurrence, metastasis, and/or all-cause death. *AKA: above-the-knee amputation, BKA: below-the-knee amputation.*

	Recurrence	Metastasis	Mean-survival (months)	All-cause death
*Type of treatment*				
Amputation	5.56% (2/36)	8.33% (3/36)	39.91 ± 27.63	20.59% (7/34)
Limb salvage	33.33% (1/3)	0 (0/2)	24 ± 21.63	0 (0/2)
*Pvalues*	0.219	0.846	0.3116	0.644

*Treatment modality*				
AKA	0 (0/16)	6.25% (1/16)	47.23 ± 31.01	33.33% (5/15)
BKA	10% (1/10)	0 (0/10)	45.78 ± 22.03	0 (0/9)
Ray amputation	0 (0/2)	0 (0/2)	10.5 ± 2.12	0 (0/2)
Foot amputation	0 (0/2)	0 (0/2)	42 ± 42.43	0 (0/2)
Hip disarticulation	0 (0/3)	33.33% (1/3)	30 ± 26.63	33.33% (1/3)
Resection	33.33% (1/3)	0 (0/2)	24 ± 21.63	0 (0/2)
Hemipelvectomy	0 (0/1)	100% (1/1)	2	100% (1/1)
Transhumeral amputation	100% (1/1)	0 (0/1)	36	0 (0/1)
Digit amputation	0 (0/1)	0 (0/1)	16	0 (0/1)

*AKA vs. BKA*				
AKA	0 (0/16)	6.25% (1/16)	47.23 ± 31.01	33.33% (5/15)
BKA	10% (1/10)	0 (0/10)	45.78 ± 22.03	0 (0/9)
*Pvalues*	0.385	0.615	0.8910	0.071

## Data Availability

All data presented in this study have been retrieved from published manuscripts cited in the references section.

## Supplementary Materials

Supplementary Materials Supplementary material 1. Quality assessment CARE checklist. Supplementary material 2. Quality assessment STROBE checklist.
