# Supplementary material for: Squamous Cell Carcinoma Arising from Chronic Osteomyelitis in the Extremities: Treatment Approach and Oncological Outcomes—A Systematic Review of the Literature
Source: J Skin Cancer. 2022 Oct 10;2022:2671420. doi: 10.1155/2022/2671420 (PMC9576437; doi:10.1155/2022/2671420)
Supplement: Supplementary Materials — Supplementary material 1. Quality assessment CARE checklist. Supplementary material 2. Quality assessment STROBE checklist. [file 2671420.f1.zip › Supplementary material 1.docx]

**Supplementary material 1**. Quality assessment CARE checklist.

|  | Title | Patient Info | Clinical Findings | Timeline | Diagnostic Assessment | Therapeutic Intervention | Follow-up & Outcomes | Discussion | Included |
| --- | --- | --- | --- | --- | --- | --- | --- | --- | --- |
| Abdul W (2017) [1] | Partly | Well | Well | Well | Well | Well | Well | Well | Yes |
| Akoh CC (2017) [3] | Partly | Well | Well | Well | Well | Well | Well | Well | Yes |
| Altunay I (2015) [7] | Well | Well | Well | Well | Well | Well | Well | Well | Yes |
| Aslan A (2020) [8] | Partly | Partly | Well | Well | Well | Well | Well | Partly | Yes |
| Bernhard K (2016) [10] | Partly | Well | Well | Well | Well | Well | Partly | Well | Yes |
| Caruso G (2016) [14] | Well | Well | Partly | Well | Well | Well | Well | Well | Yes |
| Chagou A (2020) [15] | Well | Partly | Well | Well | Well | Well | Well | Well | Yes |
| Chiao HY (2013) [17] | Partly | Well | Well | Well | Partly | Well | Well | Well | Yes |
| Hamdani M (2017) [24] | Partly | Well | Well | Well | Well | Well | Well | Well | Yes |
| Henning J (2020) [25] | Well | Well | Well | Well | Well | Well | Well | Partly | Yes |
| Hwang KT (2012) [26] | Partly | Partly | Well | Well | Well | Well | Well | Well | Yes |
| Kersh S (2010) [36] | Well | Well | Well | Well | Well | Well | Well | Well | Yes |
| Khaladj M (2015) [37] | Well | Well | Well | Well | Partly | Well | Well | Well | Yes |
| Kurihara T (2019) [40] | Well | Well | Well | Well | Partly | Well | Well | Well | Yes |
| Lack W (2010) [41] | Partly | Well | Well | Well | Well | Well | Well | Well | Yes |
| Monaco SJ (2015) [46] | Well | Well | Well | Well | Well | Well | Well | Well | Yes |
| Moyer HR (2016) [49] | Well | Well | Well | Well | Well | Well | Well | Well | Yes |
| Stanger KM (2015) [59] | Partly | Well | Well | Well | Well | Well | Well | Well | Yes |
| Steinrücken J (2012) [60] | Well | Well | Well | Well | Well | Well | Well | Well | Yes |
| Kim JH (2010) [38] | Partly | Poorly | Poorly | Poorly | Partly | Partly | Poorly | Poorly | No |
