# Supplementary material for: Squamous Cell Carcinoma Arising from Chronic Osteomyelitis in the Extremities: Treatment Approach and Oncological Outcomes—A Systematic Review of the Literature
Source: J Skin Cancer. 2022 Oct 10;2022:2671420. doi: 10.1155/2022/2671420 (PMC9576437; doi:10.1155/2022/2671420)
Supplement: Supplementary Materials — Supplementary material 1. Quality assessment CARE checklist. Supplementary material 2. Quality assessment STROBE checklist. [file 2671420.f1.zip › Supplementary material 2.docx]

**Supplementary material 2**. Quality assessment STROBE checklist.

| Study | Setting | Participants | Variables | Data Sources | Statistical Methods | Participants | Descriptive Data | Outcome Data | Main Results | Limitations | Included |
| --- | --- | --- | --- | --- | --- | --- | --- | --- | --- | --- | --- |
| Alami M (2011) [17] | Well | Well | Well | Well | Well | Partly | Well | Well | Well | Partly | Yes |
| Karasov Yesilada A (2013) [26] | Partly | Partly | Partly | Partly | Well | Partly | Well | Well | Partly | Partly | Yes |
| Li Q (2015) [32] | Well | Partly | Well | Well | Well | Partly | Well | Well | Well | Partly | Yes |
| Moura DL (2017) [34] | Partly | Partly | Partly | Well | Well | Partly | Well | Well | Well | Partly | Yes |
| Peng L (2020) [36] | Well | Partly | Well | Well | Well | Well | Well | Well | Well | Partly | Yes |
|  |  |  |  |  |  |  |  |  |  |  |  |
